# Trends in the crossover of patients in phase III oncology clinical trials in the USA

**DOI:** 10.3332/ecancer.2020.1142

**Published:** 2020-11-13

**Authors:** Justin Yeh, Shruti Gupta, Sunny J Patel, Vamsi Kota, Achuta K Guddati

**Affiliations:** 1 Medical College of Georgia, Augusta University, Augusta, GA 30909, USA; 2 Division of Hematology/Oncology, Georgia Cancer Center, Augusta University, Augusta, GA 30909, USA

**Keywords:** crossover, randomised, controlled trials, cancer

## Abstract

**Background:**

The incorporation of crossover in randomised controlled trials is accepted as an ethical obligation, especially in cancer clinical trials. The more common type of crossover is crossover allowance, which allows patients assigned to one arm to switch to another arm if there is an established benefit in the crossover arm. In contrast, crossover-designed studies involve switching patients from all arms to a different arm as part of the study design. Crossover allowance may have advantages in patient recruitment and incorporating crossover after initial positive results fulfil ethical requirements. However, crossover can also contribute to confounding major endpoints of studies, such as overall survival or the second progression-free survival interval. For this reason, it is important to investigate and identify potential trends of crossover in clinical trials testing novel therapies.

**Methods:**

Data about cancer clinical trials were extracted from clinicaltrials.gov. The search query was limited to completed phase III studies in adult populations. Location was limited to the USA. Date range extended from 1990 to 2019. Search query included the terms: cancer; completed- recruitment status; age: 18–65+ years; sex: all; location: USA; and study phase: phase 3. Studies were then excluded if they were not randomised controlled trials (RCTs) with the primary purpose of treatment and if they did not test cancer-related interventions.

**Results:**

A total of 744 clinical trials were identified. There were 459 RCTs aimed at treatment, and of those, 35 utilised crossover. The start dates of these crossover trials ranged from 1997 to 2012. Thirty studies utilised crossover allowance. Prostate, breast and gastrointestinal stromal tumour cancers were the most represented cancer types in crossover studies. Among the 30 studies, the median proportion of patients who crossed over relative to the original arm assignment ranged from 2% to 88%, with a median of 57.5%.

**Conclusions:**

The proportion of identified clinical trials with crossover compared to those without is extremely small. Crossover in clinical trials studying cancer treatment does not appear to be a widespread practice. Even though statistical approaches to mitigate confounding exist, crossover can still skew accurate reporting of the impact of experimental therapies on overall survival.

## Introduction

The term ‘crossover’ in randomised controlled trials traditionally describes a study design in which study participants are able to receive different treatments during different time periods of the study. Crossover is utilised in studies where the effects of the treatment are short-lived and do not permanently alter the process under study. They are best suited for trials that study the short-term outcomes of symptomatic, yet chronic diseases or conditions [1]. In the literature, the term ‘crossover’ can refer to two distinct processes in relation to trial design. The first and more common type is sometimes referred to as ‘treatment switching’, where patients from the control group are allowed to switch from one arm to the other arm if certain conditions are met, such as if a patient’s disease progresses and after an investigational drug is shown to have clinical superiority [2]. Most often, in this type of crossover, patients switch treatments from the standard of care to the experimental drug, but patients may also switch in the opposite direction (from experimental to standard of care) if the investigational drug is shown to be dangerous or harmful. In contrast to allowing for treatment switching, the second type of crossover is a type of study design that allows patients to act as their own controls by having patients in each arm switch to the other arms during the study period so that all patients receive all treatments included in the study [3]. Patients enrolled in these crossover-designed studies are scheduled to receive both treatments (in a two-arm study) sequentially, with the only difference between the groups being the order in which they receive the treatments. To differentiate the two types of crossover, we will use the terminology ‘crossover allowance’ and ‘crossover-designed’ to refer to studies that allow for treatment switching and studies that mandate crossover as part of their study design, respectively.

Studies with crossover allowance have many advantages and disadvantages. The incorporation of crossover allowance is often accepted as an ethical obligation, particularly in cancer randomised controlled trials (RCTs), preventing a scenario where patients are denied access to superior treatment. Crossover also increases patient recruitment [1, 4]. Patients are more likely to participate in a study where they have an opportunity to try an experimental treatment, particularly when early data may reveal it to be effective. However, crossover allowance can distort the outcomes of clinical studies. Particularly in cancer studies, crossover allowance can confound endpoints that are measured after the crossover event, such as the second progression-free survival (PFS2) interval or overall survival (OS) [1, 4–6].

Given the advantages and concerns about crossover allowance, it is important to investigate and identify potential trends of the study design in clinical trials testing new therapies. For the study of new and developmental drugs, crossover studies are extremely popular for the study of novel and developmental drugs. The utilisation of crossover in published RCTs is common as approximately a quarter of such studies have used the design in their protocols [5–7]. Crossover also seems to be becoming more common in oncology as cancer treatments become more promising. However, trends in crossover of clinical trials across all cancers are yet to be investigated. Here, we investigate the trend and proportion of crossover in phase III clinical trials for cancer treatment between the years 1990 and 2019.

## Methods

**Figure 1.**
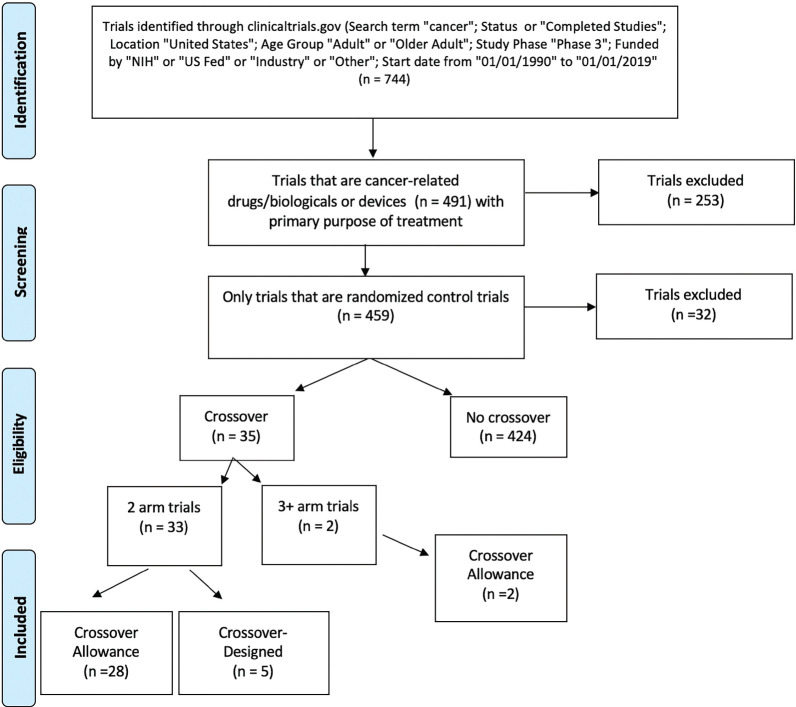
Consort diagram on research method.

Trials included in the analysis were extracted from clinicaltrials.gov with the search criteria identified in Figure 1. The search query was limited to completed phase III studies in adult populations. Inclusion criteria represented trials using the search term ‘cancer’; status active or completed studies; trials conducted on adults or older adults within the USA; phase 3 trials; trials funded by NIH, US Fed, industry, or others; and trials with a start date between 1 January 1990 and 1 January 2019. Trials were restricted to randomised control trials with the primary purpose of treatment. We defined a randomised control trial as a prospective study assessing health-care interventions in human participants who were randomly allocated to study groups. We then further narrowed to those trials that were considered cancer-related. Drug and device interventions were included as defined by clinicaltrials.gov, excluding other interventions (behavioural, radiation, procedural, etc.). Study results of all remaining trials were reviewed on clinicaltrials.gov to determine whether crossover allowance occurred. Data on crossover information from the specified trials were then extracted from clinicaltrials.gov. Crossover information was collected from data on prospective methods and trial results (preliminary or complete) from clinicaltrials.gov or PubMed. Trials designated with a crossover study design were then divided into those with two arms and those with three or more arms. Two arm trials were further divided into categories of crossover allowance and crossover-designed studies.

## Results

A total of 744 clinical trials published between 1990 and 2019 were identified. Among them, 459 studies were RCTs with the primary purpose of treatment and 35 (4.7%) either allowed crossover or were designed as a crossover study. The start dates of these crossover trials ranged from 1997 to 2012, with three trials starting within a 4-year span of 1997–2000, six trials in 2001–2004, eight trials in 2005–2008 and 18 trials in 2009–2012. Thirty-three of 35 crossover trials contained two experimental arms while the remaining two trials contained three or more experimental arms. Of the two-arm crossover trials, 28 were crossover allowance and five were crossover-designed. The two crossover trials with at least three treatment arms were both crossover allowance. Prostate, breast and gastrointestinal stromal tumour cancers were the most represented cancer types in crossover studies. A list of all 35 crossover studies can be found in Supplemental Table 1.

The proportion of patients who crossed over in the 30 studies allowing crossover (28 two-arm studies and 2 three-arm studies) was examined (Table 1). For each crossover allowance study, the number of patients who crossed over was compared with the total number of patients assigned to the original arm before crossover. In all studies except one, this proportion was calculated by obtaining the number of patients crossing over to the experimental arm (PC) and dividing by the number of patients originally assigned to the reference arm (PR). The only exception was NCT01125566, in which patients in the experimental arm crossed over to the reference arm. The median PC:PR proportion was 57.5%, with a range of 2% to 88%.

## Discussion

We found that 33 two-arm studies and two three-arm studies were either crossover allowance or crossover-designed studies. It appears that the number of crossover trials is greater in recent years, particularly after 2009, indicating that crossover is increasingly used. Despite the upward trend of crossover prevalence, these trials nevertheless represent a very small proportion of the total randomised trials we assessed. The prevalence of crossover studies in cardiology and nephrology has been reported to be 5.4% and 9.4%, respectively [8], and reviews of indexed RCTs in Medline and PubMed have reported a crossover prevalence ranging from 8.7% to 22% [9–11]. While not explicitly stated, it appears that these percentages represent crossover-designed studies. Based on the results of our analysis, the proportion of crossover trials in cancer treatment is similar to the crossover trial prevalence reported in cardiology and nephrology. However, when only examining crossover-designed studies, the proportion (0.7%) is much lower.

While crossover allowance may confer certain advantages such as providing patients with an ethical option, increased patient recruitment and improvements in outcome efficiency and statistical significance, our results indicate that it is not common practice in cancer phase III trials. Despite challenges resulting from confounding with crossover, there does not appear to be any bias in the literature against publishing results of crossover trials, as all of the crossover trials identified were successfully published.

It is important to note that the majority of crossover studies identified in this analysis were not crossover-designed, but rather allowed for crossover or treatment switching. This type of crossover is driven primarily by an ethical obligation not to withhold the treatment that has shown significant benefit. There is no ‘standard’ threshold that necessitates treatment switch, but study designs often allow patients to switch from the control to experimental arm should they experience progression of their disease, and if preliminary results indicate a statistically significant benefit of the experimental arm. In a study of afatinib plus vinorelbine in HER2+ metastatic breast cancer, the experimental arm (afatinib) was actually shown to have poorer OS compared to the control, and the independent data monitoring committee ended recruitment for the study and allowed crossover of the afatinib arm to the control after a benefit-risk assessment [12].

The relatively small number of crossover studies likely indicates that crossover has not skewed our understanding of effective therapies in the field of cancer on a macroscopic level. Within these identified studies, however, the impact of crossover on survival data is still unclear. The PC:PR proportion calculated for each study provides a perspective through which to view crossover allowance. Studies with a high PC:PR have a high proportion of patients switching treatment to the experimental arm, relative to the original number of patients starting in the reference arm. With a greater proportion of reference arm patients receiving presumably more efficacious treatment after crossover, it is reasonable to expect that the overall survival of the experimental arm would be more heavily confounded. Specifically, in cancer trials, multiple studies have shown that crossover allowance can skew study outcomes such as PFS2 and OS [4, 5, 13, 14]. This confounding is not limited to only crossover allowance, as any subsequent treatment can influence intent-to-treat estimates of the initial treatment effect [15]. As a result, intent-to-treat OS can be overestimated in the control arm, potentially underestimating the treatment benefit of the experimental arm.

Mitigating confounding from crossover can be achieved at the data analysis level through evolving statistical approaches. Rank-preserving structural failure time (RPSFT) and inverse probability of censoring weighted (IPCW) are statistical techniques that use different approaches to decrease survival bias from crossover [16]. RPSFT uses a structural model to estimate and remove the effect of crossover treatment on a patient’s survival time. IPCW operates by censoring patients at the time of crossover and relying on data from similar patients who did not crossover to estimate survival. These methods have been tested and applied [17–20] but nevertheless have their own limitations [16].

## Strengths and limitations

There are several important strengths and limitations of this study. Strengths include our search of clinicaltrials.gov during the study period to find studies that met our search conditions. We examined at a census of all randomised controlled trials and then narrowed our searches to trials of significance for this study. Moreover, we looked at a large sample size for the study and thus are confident that the results provide an accurate depiction of the trends in crossover. The main limitations of this study are due to the singular registry for clinical trials used in the search process, as well as including only trials with US locations. While clinicaltrials.gov is the largest clinical trials database, other trials found only in other sources were not included; while some international trials were not included in the analysis, larger multinational trials that included US involvement were captured by our search. For example, large phase III breast cancer trials in the early 2000s, such as MA.17 (NCT00003140), HERA (NCT00045032) and BIG 1-98 (NCT00004205), all included allowance for crossover, but did not have trial sites within the US [21–23]. In the HERA study, 888 of 1,697 patients (52.3%) in the observation arm crossed over to trastuzumab, similar to the median PC:PR found in US studies. Further investigation may examine the prevalence of crossover in non-US-based trials and whether crossover allowance is more or less common, as well as the cancer types that contain a relatively higher number of crossover studies internationally.

## Conclusions

Crossover study designs have been a popular option in randomised clinical trials. However, the prevalence of crossover in cancer phase III trials is minimal but increasing. We hope that these results will prove helpful to investigators designing, managing and interpreting clinical trials involving crossover for cancer drugs. The increasingly sophisticated statistical methods mitigating crossover bias are necessary now, more than ever, given the higher number of cancer RCTs with crossover allowance and persisting ethical obligation to patients.

## Funding statement

The authors declare that there was no funding for this study.

## Conflicts of interest

None.

## Figures and Tables

**Table 1.** Studies allowing for treatment crossover.

Condition	NCT number	Patients in exp. arm	Patients in ref. arm (PR)	Crossover patients (PC)	PC/PR	Primary experimental intervention
**Breast cancer**						
	NCT00022672	103	104	58	56%	Trastuzumab
	NCT00435409	221	221	78	35%	Sunitinib
	NCT01125566	339	169	^a^75	22%	Afatinib
**Carcinoid tumour**						
	NCT00412061	216	213	170	80%	Everolimus
**Colorectal cancer**						
	NCT01103323	505	255	4	2%	Regorafenib
**Gastrointestinal stromal tumours**						
	NCT00075218	243	118	103	87%	Sunitinib
	NCT00471328	165	83	67	81%	Nilotinib
	NCT01271712	133	66	NP	--	Regorafenib
**Hepatocellular Carcinoma**						
	NCT00105443	299	303	47	16%	Sorafenib
**Melanoma**						
	NCT01006980	337	338	84	25%	Vemurafenib
	NCT01227889	187	63	37	59%	Dabrafenib
	NCT01245062	214	108	70	65%	Trametinib
**Prostate cancer**						
	NCT00974311	800	399	50	13%	Enzalutamide
	NCT01212991	872	845	234	28%	Enzalutamide
	NCT00699751	614	307	26	8%	Radium-223 dichloride
	NCT00887198	546	542	93	17%	Abiraterone acetate
	NCT00451958	210	204	135	66%	Degarelix
**Renal cell carcinoma**						
	NCT00073307	451	452	299	66%	Sorafenib
	NCT00410124	277	139	111	80%	Everolimus
**Subependymal giant cell astrocytoma**						
	NCT00789828	78	39	33	85%	Everolimus
**Thyroid cancer**						
	NCT00984282	207	210	161	77%	Sorafenib
	NCT01321554	261	131	109	83%	Lenvatinib
**Chronic lymphocytic leukaemia**						
	NCT01010061	238	118	NR		Obinutuzumab
**Cutaneous T-cell lymphoma**						
	NCT01728805	186	186	136	73%	Mogamulizumab
**Diffuse large B-cell lymphoma**						
	NCT01197560	54	57	29	51%	Lenalidomide
**Mantle cell lymphoma**						
	NCT00117598	57	56	4	7%	Temsirolimus
			56	3	5%	
**Multiple myeloma**						
	NCT00064038	100	98	42	43%	Lenalidomide
	NCT01311687	302	153	11	7%	Pomalidomide
**Myelodysplastic syndromes**						
	NCT00003138	57	61	26	43%	Erythropoietin + Filgrastim
**Myeloproliferative neoplasms**						
	NCT00952289	155	154	111	72%	Ruxolitinib

a Patients crossed over from experimental arm to reference arm
